# Adaptation and Codon-Usage Preference of Apple and Pear-Infecting Apple Stem Grooving Viruses

**DOI:** 10.3390/microorganisms9061111

**Published:** 2021-05-21

**Authors:** Jaedeok Kim, Aamir Lal, Eui-Joon Kil, Hae-Ryun Kwak, Hwan-Su Yoon, Hong-Soo Choi, Mikyeong Kim, Muhammad Ali, Sukchan Lee

**Affiliations:** 1 Department of Integrative Biotechnology, Sungkyunkwan University, Suwon 16419, Korea; elhanan06@korea.kr (J.K.); aamirchaudhary43@gmail.com (A.L.); viruskil@anu.ac.kr (E.-J.K.); 2 Incheon International Airport Regional Office, Animal and Plant Quarantine Agency, Seoul 22382, Korea; 3 Department of Plant Medicals, Andong National University, Andong 36729, Korea; 4 Crop Protection Division, National Institute of Agricultural Sciences, Rural Development Administration, Wanju 55365, Korea; hrkwakhahn@korea.kr (H.-R.K.); hschoi@korea.kr (H.-S.C.); 5 Department of Biological Science, Sungkyunkwan University, Suwon 16419, Korea; hsyoon2011@skku.edu; 6 College of Agriculture, Life and Environment Sciences, Chungbuk National University, Cheongju 28644, Korea; 7 Department of Life Sciences, School of Science, University of Management and Technology (UMT), Johar Town, Lahore 54770, Pakistan

**Keywords:** ASGV, coat protein, host adaptation, RNA virus, virus evolution

## Abstract

Apple stem grooving virus (ASGV; genus *Capillovirus*) is an economically important virus. It has an approx. 6.5 kb, monopartite, linear, positive-sense, single-stranded RNA genome. The present study includes identification of 24 isolates—13 isolates from apple (*Pyrus malus* L.) and 11 isolates from pear (*Pyrus communis* L.)—from different agricultural fields in South Korea. The coat protein (CP) gene of the corresponding 23 isolates were amplified, sequenced, and analyzed. The CP sequences showed phylogenetic separation based on their host species, and not on the geography, indicating host adaptation. Further analysis showed that the ASGV isolated in this study followed host adaptation influenced and preferred by the host codon-usage.

## 1. Introduction

An understanding of the molecular evolution of viruses is essential to design knowledge-based control strategies. Relatively fewer studies are available on plant viruses covering sequence origin, mutation rates, selection pressure, reassortment, and recombination [1] Previous studies suggest point-mutations, recombination, and reassortments as major driving forces in evolution for the natural selection. The mechanisms of adaptation and selections contributing a virus fitness are yet to be explored exactly [2].

The adaptation of viruses to novel hosts is a general evolutionary phenomenon of invasion and adaptation to a new niche. The new host may present challenges at the level of viral entry into cells, replication, and transmission/exit. Similarly, the new host may encode different/stringent defense mechanisms, which the newly invading virus has to overcome. Only a small minority of the initial pool of viral genotypes may survive these hurdles and that subsequent adaptation will likely lead to improved adaptation [3].

Parallel evolution, i.e., independent evolution of similar or identical features in distinct lineages subject to similar selection pressures [4], were reported extensively in both natural and experimental populations of microbes—most often viruses [4,5,6], bacteria [7], yeast [8], and protozoa [9]. Differences in the founding genotypes may result in divergent evolutionary trajectories [10,11]. It is, therefore, important to know the dynamics of evolution that could be predicted through parallel evolution.

*Apple stem grooving virus* (type species, genus *Capillovirus*) is an economically important virus of pome fruits and citrus, particularly plant species of *Malus* and *Pyrus* [12]. A virion of apple stem grooving virus (ASGV) contains monopartite, linear single-strand positive-sense 6.7–7.4 kb RNA genome. As host plants are often asymptomatic, the virus survives long enough, unnoticed, to adapt to woody plant hosts.

Although ASGV is latent in most commercial rootstock–scion combinations, serious disease burden is evident in apples grown on *Malus sieboldii* rootstocks in Japan [13]. In pear, ASGV claimed black necrotic leaf spot disease where a decline up to 50% has been estimated [14].

Looking into the importance of the virus, we assessed the dynamics of evolution to examine the molecular basis of adaptation in a viral population under host-derived natural selection in apple and pear plants.

## 2. Materials and Methods

### 2.1. Sample Collection

**Figure 1:**
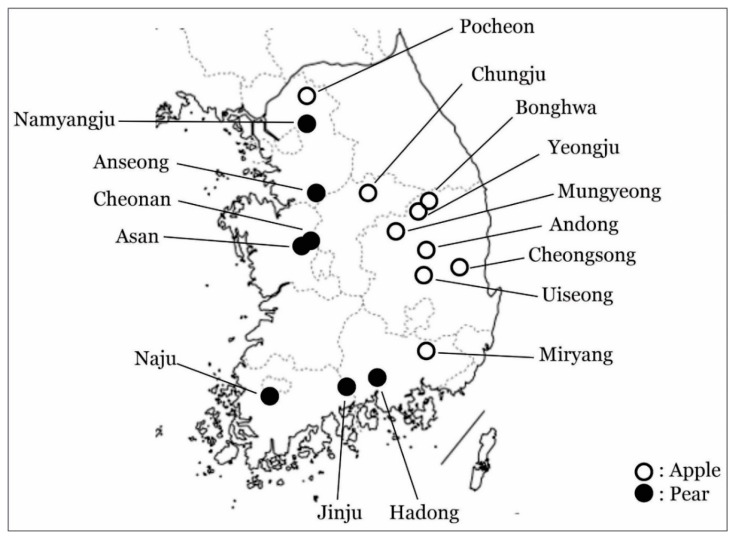
Geography of plant samples collected in this study for apple stem grooving virus amplification.

Apple and pear plant samples were collected from major agricultural areas in South Korea (Figure 1) in the years 2008 and 2009. Petal and leaf tissues were collected from randomly chosen trees in each field. All collected tissues were stored at −80 °C until plant total RNA was extracted using the Easy-Spin™ (Intron Co., Seongnam, Korea) RNA extraction kit following manufacturer’s instructions.

### 2.2. cDNA Amplification

Primers for the ASGV coat protein (CP) gene were designed as: *forward primer* 5′-AGG CWA TCA GCG GCT GTG-3′ and *reverse primer* 5′-AGA GTG GAC AAA CTC TAG ACT CTA GAA A-3′. A reference sequence (GenBank AB004063) was used to design the primers. The cDNA synthesis was carried out in a 5 µL reaction mixture. Briefly, 0.1 ng to 5 µg plant total RNA, 20 pmol reverse primer was taken. The volume was made up to 3 µL with DEPC-treated water. The mixture was incubated for 5 min at 65 °C, followed by chilling on ice. Before incubating reaction mixture for 60 min at 42 °C, 1× reaction buffer (Promega Co., Madison, WI, USA), 1 mM final concentration of dNTP mix and 1 unit of AMV Reverse Transcriptase was added. The product of reverse transcription was directly used in downstream PCR or stored at −20 °C.

A final volume of 25 µL reaction mixture—comprised of 1× Taq buffer, 200 µM dNTPs mix, 1.5 mM MgCl_2_, 10 pM of each forward and reverse primer, 1 unit of Taq DNA Polymerase (Cat# M7660, Promega Co.), and 5 µL of reverse transcription product—were incubated in a thermal cycler. The incubation program was executed as—denaturation for 3 min at 94 °C, followed by amplification for 35 cycles (94 °C for 30 s, 55 °C for 30 s, and 72 °C accordingly as 1 min per kb amplicon). The amplification was followed with a final extension of 10 min at 72 °C. The PCR product was visualized on 1% agarose gel.

### 2.3. Phylogenetic Analysis

Amplified PCR amplicons were sequenced in both orientations, assembled and analyzed. The sequences were then submitted to the GenBank (Table S1). The ASGV sequences identified in this study were aligned with the previously available sequences in the databases. The evolutionary history was inferred from the nucleotide sequences using the MUSCLE method based on K2+G model (Figure 4). The bootstrap consensus tree was inferred using 100 replicates representing the evolutionary history. The tree is drawn to scale, with branch lengths measured in the number of substitutions per site.

Nucleotide sequences were translated into amino acid sequences. The evolutionary history of the amino acid sequences was inferred using the MUSCLE method, based on the JTT+G best-fit-model (Figure 5). The bootstrap consensus tree was inferred from 100 replicates representing the evolutionary history. All other options followed as described above.

### 2.4. Protein Structure Prediction

Multi-aligned amino acids sequences were analyzed using Geneious Pro 8 (Biomatters Co., Auckland, New Zealand). Secondary structures were predicted using the original Garnier Osguthorpe Robson algorithm (GOR I) provided by the EMBOSS suite embedded in Geneious Pro 8. Tertiary structures were predicted and modeled using the i-TASSER server [15].

### 2.5. Codon-by-Codon Behavior Analysis for Synonymous and Non-Synonymous Substitutions

Among the sequences identified in this study, only fully characterized ASGV CP sequences were selected. These sequences were analyzed to determine the cumulative behavior of sequence variation in a codon-by-codon manner with the synonymous and non-synonymous analysis program [16]. 

### 2.6. Codon-by-Codon Maximum Likelihood Analysis of Natural Selection

For each codon, estimates of inferred synonymous (s) and nonsynonymous (n) substitutions were presented along with the numbers of sites that were estimated to be synonymous (S) and nonsynonymous (N). These estimates were produced using joint maximum likelihood reconstructions of ancestral states under a Muse-Gaut model of codon substitution and Tamura-Nei model of nucleotide substitution. For estimating ML values, a tree topology was automatically computed. The test statistic dN-dS was used to detect codons that had undergone positive selection; where, dS is the number of synonymous substitutions per site (s/S) and dN is the number of nonsynonymous substitutions per site (n/N). The analysis involved 25 Korean ASGV isolates. The codon positions included were 1st + 2nd + 3rd + Noncoding. All positions containing gaps and missing data were eliminated. There were 675 positions in the final dataset. Evolutionary analyses were conducted in MEGA6 [17].

### 2.7. Preferred Codon Usage of ASGV CP Gene

Codon usage of ASGV CP genes was analyzed using Geneious Pro 8 with multi-aligned nucleotide and the corresponding amino acid sequences. Codon usage of ASGV was analyzed in two ways. First, a correspondence analysis for codon usage was performed for all Chinese and Korean isolates. Second, codon usage frequencies among apple and pear isolates were processed on the codon usage database and codon usage analyzer [18].

## 3. Results

A total of 24 amplicons were sequenced in both orientations. Sequencing results confirmed ASGV sequences. The sequences were 710–711 nucleotides long covering complete coat protein gene coding sequence (CDS) except the stop codon (i.e., TAG and, in some cases, a T before the stop codon). The sequences were then submitted to the GenBank (JN792471 to JN792494).

### 3.1. Phylogenetic Analysis of ASGV Showed Host-Specificity

To determine ASGV host-specificity, 56 CP sequences, including 24 isolates identified in this study (i.e., 13 isolates from apple, 11 isolates from pear) and a former Korean isolate (GenBank AY596172) from pear, identified prior to this study, were used. Phylogenetic analysis based on nucleotide sequences revealed clustering of clear distinct patterns corresponding to their isolation hosts. Additionally, intermediate groups were also observed (Figure 4).

Most ASGV isolates worldwide were categorized in clade 4. Countries from which the isolates were reported are the Latvia, Serbia, and Turkey in Europe; China, India, and Korea in Asia; and Brazil in the Americas. Regardless of the location, ASGV were easily distinguished by their isolation hosts. Similar to the clade 4, ASGV isolates from China in intermediate^2^ was also separated to their geography.

**Figure 2:**
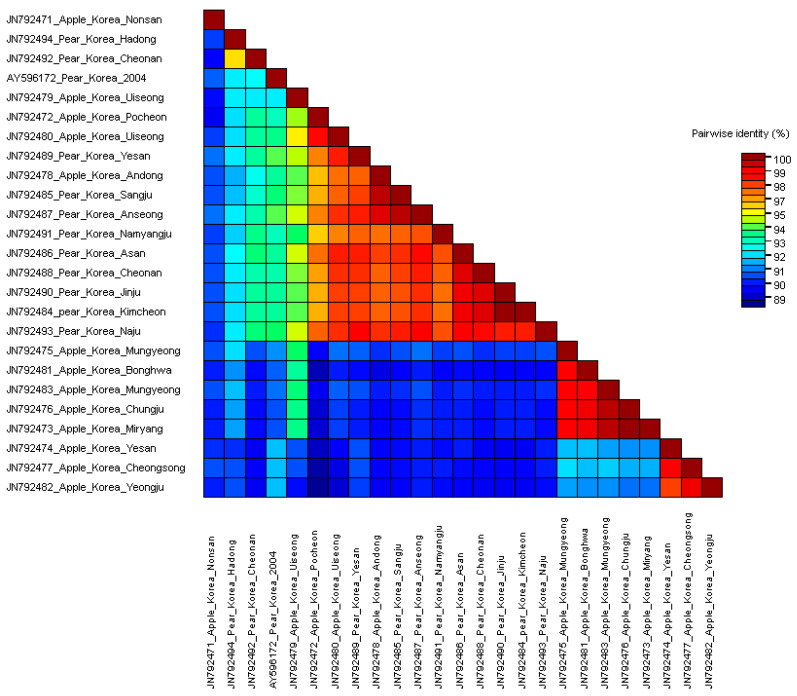
Apple stem grooving virus (ASGV) coat protein gene nucleotide sequence identities. MUSCLE alignment was used implemented in Sequence demarcation tool (SDTV1.2).

Interestingly, clade 3 contains Korean isolates identified on apple trees. Along with this, the clade 3 contains a Japanese isolate identified on apple trees. Most importantly, all isolates collected from pear trees from the distant regions were categorized in intermediate clade 1, referred to as the Korean pear group, and the clade 2. These distant regions included Anseong (GenBank JN792487), Asan (GenBank JN792486), Cheonan (GenBank JN792488), Kimcheon (GenBank JN792484), Naju (GenBank JN792493), Namyangju (GenBank JN792491), and Sangju (GenBank JN792485). To conclude, the distinctly located isolates showed high identities based on their isolation host—e.g., the two pear isolates Cheonan (GenBank JN792492) and Hadong (GenBank JN792494) showed 95.6% nucleotide identities and clustered in the clade 2 (Figure 2, Figure 4 and Figure S1).

**Figure 3:**
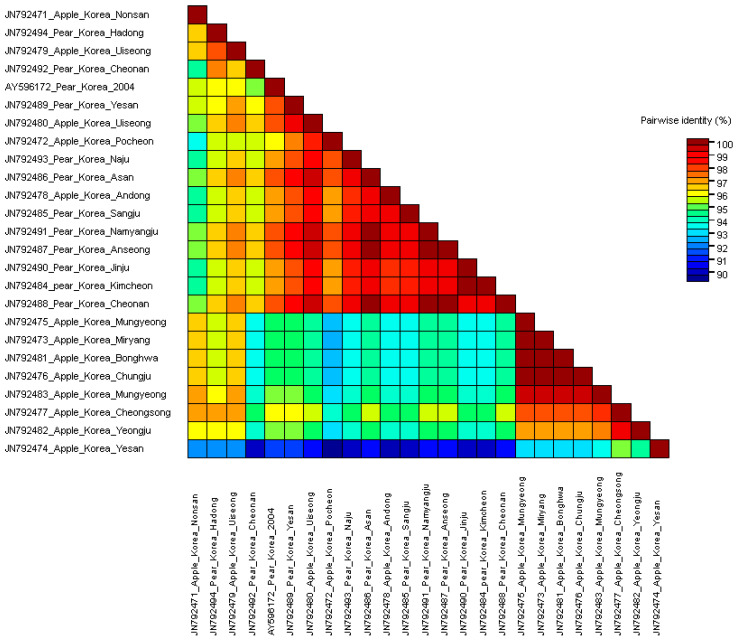
Apple stem grooving virus (ASGV) coat protein amino acid sequence identities. MUSCLE alignment was used implemented in Sequence demarcation tool (SDTV1.2).

**Figure 4:**
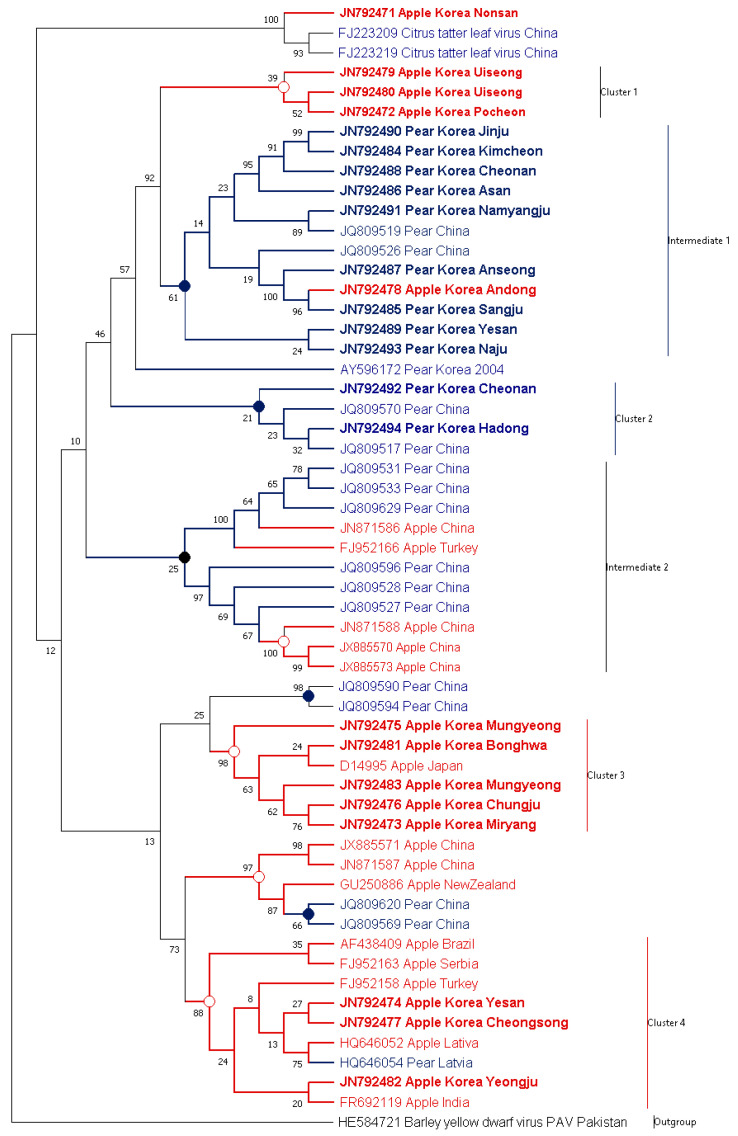
Phylogenetic analysis of apple stem grooving virus (ASGV) coat protein gene nucleotide. The tree is divided into distinct and intermediate clades. The Korean isolates identified in this study are shown with bold. Taxon names are color coded by isolation host (red for apple and blue for pear). The bootstrap support value is displayed on each branch node. The tree was generated using Maximum Likelihood with K2+G best-fit-model implemented in MEGAX.

**Figure 5:**
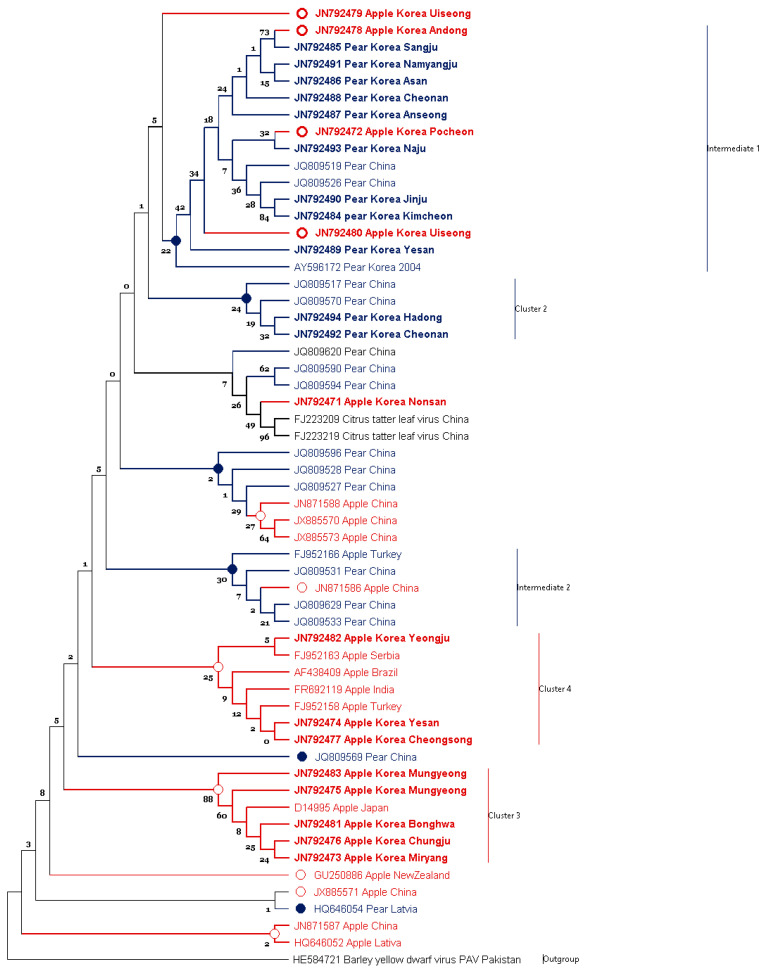
Phylogenetic analysis of apple stem grooving virus (ASGV) coat protein amino acid sequences. The tree was divided into distinct and intermediate clades. The Korean isolates identified in this study are shown with bold. Taxon names are color coded by isolation host (red for apple and blue for pear). The bootstrap support value is displayed on each branch node. The tree was generated using Maximum Likelihood with JTT+G best-fit-model implemented in MEGAX.

**Figure 6:**
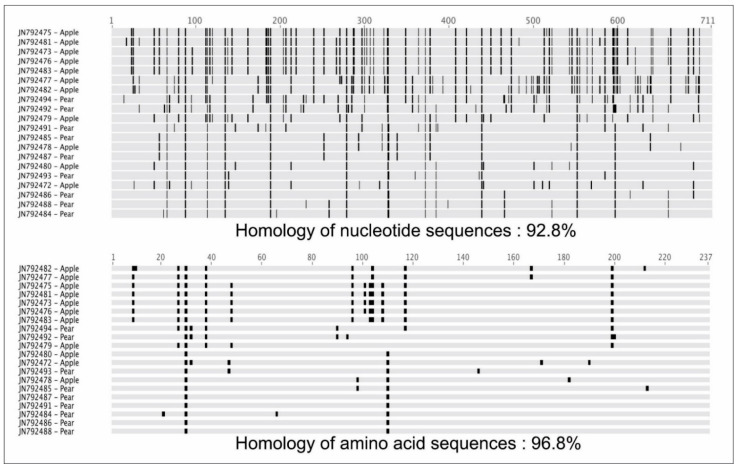
Multi-aligned sequence comparisons between nucleotides and amino acids. Identities of nucleotide and amino acid sequences were different from each other. Black highlighted positions represent differences between residues and the consensus sequence. Nucleotide sequences showed various residue substitutions coding for the same amino acids.

The isolates from the pear group exhibited significant differences in nucleotide sequence identity up to 10% against the apple isolates (Figure 6). Four apple isolates cluster into clade 1, GenBank JN792472 (Pochen), JN792479 (Uiseong), and JN792480 (Uiseong) (Figure 4). Although the pear isolates were collected from far flung distant areas, the pear isolates showed close relationship with each other and were distinguished from the apple isolates (Figure 1 and Figure 4). Isolates from apple and pear identified from the same or nearby regions did not show much relevance. For example, the accession with GenBank JN792492 from Yeongju in pear and JN792478 from Andong in apple were collected from nearby regions were clustered in distinct clades 2 and intermediate^1^ clade, respectively (Figures 4 and 5). Similarly, the two accessions from Yesan (GenBank JN792474 (apple) and JN792489 (pear)) shared only 90.0% nucleotide and 91.5% amino acid identities with each other (Figures S1 and S2). The distribution of the Yesan isolates into distinct clades, i.e., clade 4 and intermediate^1^ clade (Figures 4 and 5) indicates the isolation host specificity (also evident from Figure 2 and Figure 3).

The CP sequences were translated to amino acid sequences and aligned using the MUSCLE algorithm. The phylogenetic analysis showed that the amino acids sequences were similar to the nucleotide sequences in context with host relevance (Figure 5). Contrary to the nucleotide sequence tree, the amino acid sequence tree showed low bootstrap support, which may mean that the amino acid sequence variation was lower than the nucleotide sequences.

Both phylogenies followed the same clustering trend (Figure 4 and Figure 5). The Korean apple isolates had Gln^9^, Ala^27^, Leu^30^, Gly^38^, Lys^48^, Lys^96^, Glu^103^, Arg^104^, Ala^108^, Ser^110^, Ile^117^, and Arg^199^ residues, whereas the Korean pear isolates had Leu^9^, Gly^27^, Ser^38^, Arg^48^, Arg^96^, Glu^103^, Lys^104^, Glu^108^, Met^110^, Val^117^, and Lys^199^. The results of the amino acid sequence analysis showed differences between the apple and pear isolates in terms of amino acids residues. These changes suggest that the differences could be expressed in their structure. To explore these differences, secondary structures of the peptide chains were predicted.

The secondary structure predictions showed categorization corresponding to the phylogenetic groupings of nucleotide and amino acid sequences. Representative schematic diagrams of the predicted secondary structures are shown in Figure S4. Amino acid residue variations are reflected in the secondary structure prediction. Residue variations in the apple isolate group included Gln^9^, Ala^27^, Leu^30^, Gly^38^, Lys^48^, Lys^96^, Glu^103^, Arg^104^, Ala^108^, Ser^110^, Ile^117^, and Arg^199^_,_ compared to the pear isolates and reflected helix length, strand and coil structure, and appearance of the beta-turn.

Among these variations, the most distinguishable locus was located between the 100th and 130th amino acids. At this locus, the apple isolates had shorter helix structure than the pear isolates, due to a change in residues at the 110th position; the pear isolates had a methionine residue instead of serine as in the apple isolates. Similarly, Lys^198^ residue substitution in the pear isolate group for Arg^198^ in the apple isolate group resulted in clear differences in the secondary structure. This residue difference changed the length of the helix structure and appearance of the beta-turn structure (Figure S4).

Furthermore, all these variations in the secondary structure are reflected in the tertiary structure prediction model. From the phylogenetic analyses based on the amino acid sequences, a total of eight isolates were selected and the tertiary structures were determined for comparisons between the apple and pear groups. From the pear group, the isolates GenBank JN792486, JN792484, JN79247, JN792485, and JN792472 were selected and from the apple group, the isolates GenBank JN792477, JN 792483, and JN792475 were selected (Figure S6). These were representative of the subgroups observed in the phylogenetic tree of the amino acid sequences.

The predicted structures differed significantly from each other. In particular, GenBank JN792486 and JN792484 were the most divergent among the eight isolates. Their structure predictions were quite different from the structure of the apple isolate group; the N-terminal region was folded in a different direction compared with that in the apple isolate group. In contrast, three other sequences had similar structures to the apple isolate group. In addition, all isolates showed structural differences corresponding to their clustering in the amino acid phylogenetic tree (Figure S2).

### 3.2. Comparisons of Nucleotide and Protein Sequences

**Figure 7:**
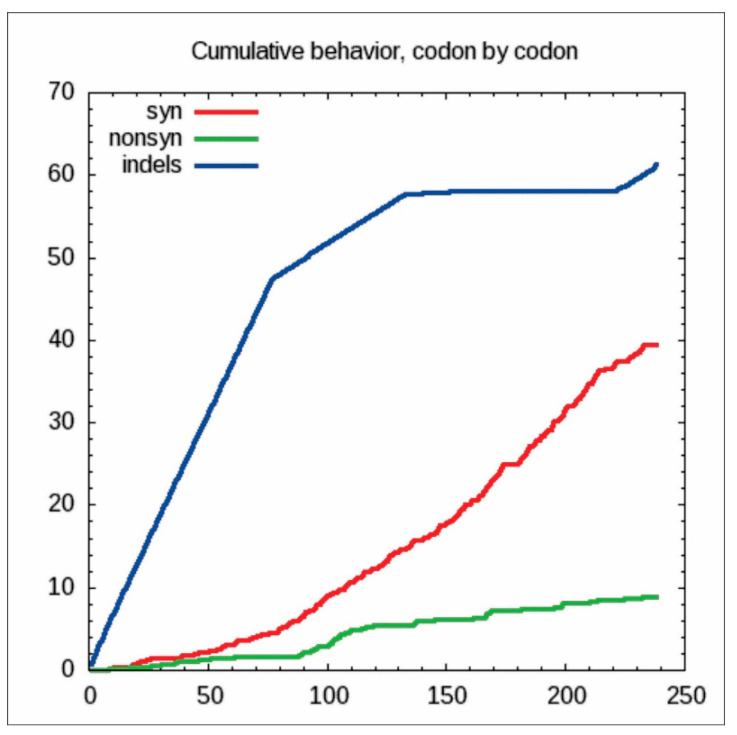
Cumulative behavior (codon-by-codon) of Korean isolates of *Apple stem grooving virus*.

Few differences were observed when phylogenetic trees based on amino acid and nucleotide sequences were compared. For instance, the maximum nucleotide and amino acid sequence identities of Korean ASGV isolates were 92.8% and 96.4%, respectively (Figure 6). As predicted, the amino acid sequence alignment showed fewer differences compared to the nucleotide sequences. The cumulative codon-by-codon behavior analysis for synonymous and non-synonymous substitutions showed a higher rate of synonymous (dS) than non-synonymous (dN) substitutions indicating purifying selection (Figure 7).

### 3.3. The CP Gene Corresponds with the Host Codon Usage Frequency

**Figure 8:**
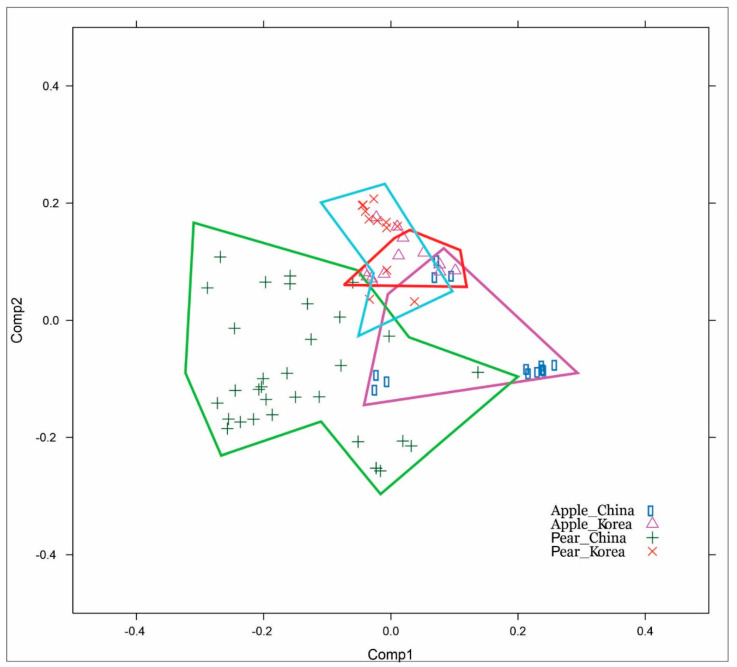
Correspondence analysis with codon usage of apple stem grooving virus (ASGV) coat protein gene from Korea and China. Codon usage of ASGV corresponded with the host and country of collection. Region with red boundary line indicates in majority the Korean ASGV collected from apple. The area with a blue boundary shows Korean ASGV collected from pear. Region with a pink boundary indicates Chinese ASGV collected from apple. Region with green circumference indicates Chinese ASGV collected from pear.

The differences in the nucleotide sequences might better be explained by variation in codon usage. Specifically, Chinese and Korean isolates were clearly separated according to their hosts. More precisely, codon usage of the ASGV CP gene from Korea and China illustrated that isolates could be distinguished by the isolation country as well (Figure 8).

**Figure 9:**
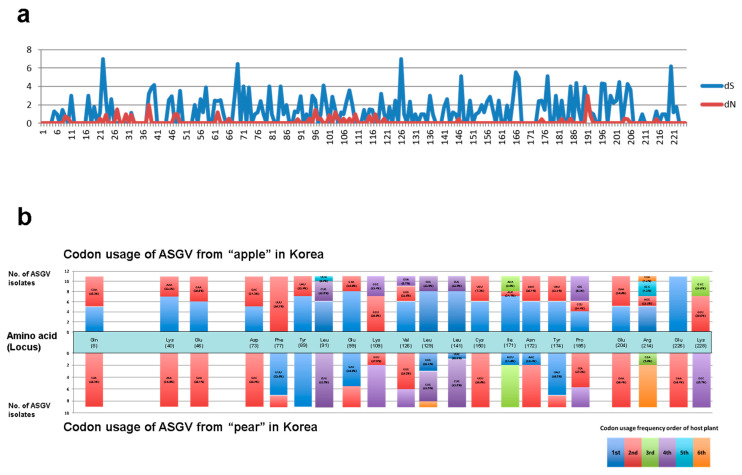
Codon usage analysis of Korean apple stem grooving virus (ASGV) coat protein gene. (**a**) Maximum likelihood analysis of natural selection codon-by-codon. The analysis involved 25 nucleotide sequences with 675 positions of ASGV codons. Most nucleotide substitutions were synonymous over the entire coat protein gene of Korean ASGV. (**b**) Analysis of ASGV codon usage at loci of synonymous substitutions compared with host preferred codon usage frequency. Substitutions of virus gene codons tended to follow the preferred codon usage of the isolated host.

For the Korean ASGV isolates, the codon usage ratio was examined codon-by-codon via a maximum likelihood analysis of natural selection. Codon usage variations appeared throughout the entire sequence of the CP gene. Specifically, these variations were not relevant to their genomic distance on the CP gene. The dS rate was higher than the dN rate for all codons among the 237 residues (Figure 9a). To characterize codon usage variation tendencies, the usage of every codon between isolates obtained from apple and pear were compared on the multiple sequence alignment. We analyzed 208 dN among a total of 237 residues for the Korean ASGV CP gene. The codon usage analysis showed variations between the CP gene of apple and pear isolates at 95 loci among 18 amino acids. Moreover, apple isolates showed more variation (showing more diversity) in codon usage than the pear isolates. Apple isolates showed variation at 79 loci as compared to 56 loci in pear isolates. Both the variation and variety of the codon usage was high in the apple isolates when compared with pear isolates. For example, there was more codon usage variety for glutamine residues among apple isolates, whereas the codon usage was conserved in pear isolates. Similarly, codon usage variations for other amino acids were more highly conserved in pear isolates than apple isolates. Except for those variants, most codon use was similar for the entire CP gene (Figure 9b).

Among these variants, codon usages of apple isolates seem to follow the codon usage frequency of the apple host at 21 amino acid residues loci (Figure 9b). In pear isolates, the codon for Cys^150^ was UGU only, whereas among the 13 apple isolates, six were UGC and five were UGU. In apple, the UGC codon was 3.4% more frequent than UGU. In pear isolates, for Asp^73^, the codon was GAC, whereas among the 13 apple isolates five were GAU and six were GAC. The frequency of GAG was 6% higher than that of GAC in apple isolates. Codon usages for Glu were tended to follow the apple gene codon usage. Compared to pear isolates, apple isolates were more likely to follow host codon usage frequency. Codon usage frequency for Glu was similar across pear isolates, with only a 0.2% difference. However, GAG was 6.1% more frequent than GAA in apple isolates. In apple isolates, Glu^46^ was represented by the GAG and GAA codons six and five times, respectively, whereas in pear isolates, only the GAA codon was observed. Similarly, in apple isolates, for Glu^99^, GAG and GAA appeared eight and three times, respectively, whereas, in pear isolates, they appeared one and eight times, respectively. At the 205th locus, in apple isolates, GAG and GAA were observed five and six times, respectively, whereas among pear isolates, only the GAA codon was observed. At the 226th locus, GAG was observed only in apple isolates, whereas GAA was exclusively observed in pear isolates.

Codon usage variations in apple isolates for Gly^109^, Gly^228^, Ile^171^, Lys^40^, Leu^91^, Leu^129^, Leu^141^, Pro^185^, Arg^214^, Val^126^, Tyr^89^, and Tyr^174^ were similar to the apple host codon usage. The codon usage frequency was similar for the similar amino acid, for instance, the codon frequency of apple for glutamine represented a difference of 1 percent only. Furthermore, analysis of Chinese ASGV isolates showed similar trend. These results indicate that the codon usage follows host codon usage frequency (Figure S3).

## 4. Discussion

Mutations, recombinations, and reassortments are the major driving forces explored extensively in plant virus evolution [2]. Even single amino acid mutations result in dramatic changes in the biological properties of viruses, such as symptom severity and systemic infectivity. Increased symptom severity in tolerant zucchini cultivars attributed to the point mutations in Zucchini yellow mosaic virus sequence [19]. Similarly, the loss and recovery of the systemic infectivity to cucumber mosaic virus in squash linked to a single amino acid mutation in the CP (Thompson et al., 2006).

In a similar way, recombination of viral genomes could also change the biological properties of viruses. RNA-RNA homologous recombination and virus evolution studies have a long history [20]. Most studies have examined phylogenetic relationships and viral genome recombination [1,21,22]. Furthermore, the effects of viral genome recombination have been studied using artificial recombinant genomes to demonstrate the loss and recovery of viral infectivity [23]. Genome reassortment is yet another driving force of evolution in multi-partite viruses. Studies on plant virus genome reassortment have mostly been conducted with the cucumber mosaic virus mainly due its split genome organization, i.e., three-segments [24,25].

In this study, 24 ASGV CP isolates (i.e., 13 isolates from apple and 11 isolates from pear) were amplified and analyzed along with 01 former Korean isolates from pear. The amplicons were sequenced in both orientations. Mix infection of a sample with different isolates remains a major possibility; however, was not studied. The sequences were then analyzed to identify the forces driving plant virus evolution. Phylogenetic analyses showed interesting categorizations of the ASGV CP gene. All the analyzed isolates could be categorized in two groups through their corresponding hosts, pear or apple. First, recombinations among virus CP gene were analyzed. All apple and pear isolates were infected with different viruses, Apple chlorotic leaf spot virus (ACLSV) and Apple stem pitting virus, in different combinations. These viruses were classified in the same virus family, *Flexiviridae* [26,27]. To determine the relationship between recombination and phylogenetic categorization, we analyzed recombination in ASGV CP genes with co-infected virus genes. According to the viral infection status, most apple isolates were co-infected with ACLSV, whereas pear isolates were not. However, we did not detect any recombination event reliable/acceptable among viral species (data not shown).

Second, to explain the categories detected, we took clues from studies regarding host adaptation of viruses. Previous host adaptation studies have examined different viral species with various experimental hosts. Ohshima et al. (2002) analyzed the molecular variability of 66 isolates of turnip mosaic virus (TuMV) isolated from several hosts. In TuMV, phylogenetic categorization showed host and geographical differences. Sacristán et al. (2005) assayed the infectivity of cucumber mosaic virus during the 10th passage of virus inoculation to examine host adaptation. After 10 passages of inoculation, viral accumulation significantly increased on newly introduced hosts. Similarly, viruses on woody plants showed similar phenomena. Symptom severity changed when the viruses were introduced to different hosts [28,29].

Molecular analysis has been conducted to characterize the biological properties of viruses that have adapted to the introduced hosts. RNA genome sequence variations were analyzed to determine the evolutionary history of viruses through the host passages and examine changes in amino acid sequence substitutions [30,31].

All sequences were categorized into two major groups accordingly as per their hosts. The bifurcation is evident both in nucleotides and amino acids sequence analysis. These groups showed significant differences in predicted CP structures. Torres et al. (2005) and Pinel-Galzi et al. (2007) previously suggested selective forces and pathways of virus evolution. The present genome variation analysis elucidated the direction of the virus evolution pathway. Together, these studies suggested that genomic evolution is still occurring, and that the direction can be determined. Similar to that in previous studies, we found evidence of evolution in the ASGV CP, with phylogenetic differences corresponding to the hosts in both nucleotide and amino acid levels (Figure 4 and Figure 5). The synonymous microevolution of viral and host genomes suggests that viral genomic evolution is related to adaptation to newly introduced hosts. As Taq DNA polymerase (Cat# M7660, Promega Co.) was used for amplification, there might be some un-intended errors/mutations introduced in the amplicons due to lack of the polymerase proof reading activity. However, a trend of host-related adaption while amplifying multiple samples indicate a minimal impact of the PCR induced mutations.

The phylogenies based on nucleotide and amino acid sequences showed interesting variations. The variations were mostly caused by silent mutations in the genome as an index of wobble hypothesis. All codon usage differences followed the phylogenetic categorization corresponding to their isolated hosts. To explain the relationship between phylogenetic categorization and hosts, viral gene codon usages were compared with host codon usage frequencies. We found that the codon usage variations followed the host codon usage frequency. Most variations that agreed with host codon usage appeared in isolates from apple. For 20 loci of the ASGV CP, more than 5 isolates from apple had higher frequencies of codon similarity to the hosts, whereas pear isolates had lower frequencies of codon similarity. Pear isolates also followed the higher frequency host codons at least at two loci.

In plant evolution studies, codon usage has not been considered an important selective force [32]. However, the dicots- and monocots-infecting sobemoviruses were categorized in the same groups [10]. Nevertheless, studies of plant virus evolution that examine codon usage were not considered useful to better understand host specificity. Unlike studies of plant viruses, in animal viruses, codon usage is considered an important factor in phylogenetic relationship along with base composition [33]. Additional studies of animal viruses suggested that relationships in codon usage between the host and the virus are driving forces of virus evolution [34]. Moreover, viral protein codon adaptation to the host, as observed in viral proteins, does not require host-specific recognition [35].

We detected host adaptation with synonymous changes in the CP gene among apple isolates of ASGV. In addition, codon usage for the ASGV CP gene was conserved in isolates from pear compared with those from apple. A study on the evolutionary trajectory of *Turnip mosaic virus* reported higher nucleotide diversity among viral genomes that had been introduced to new hosts [3]. Codon usage among pear isolates was conserved, unlike the high frequency of codon variations of pear. Conserved codon usage followed tendencies for low frequency codon use of hosts. Our results are similar to the analyses of codon usage bias of Antoniw and Adams (2003). Codon usage variation among apple isolates may, therefore, constitute evidence of ASGV adaptation to hosts. As far as protein structure analysis is concerned, the structural variability was evident in the 3D conformations of the two groups of CP (Supplementary Figure S3).

Based on the ASGV CP gene, host preference/adaptation is evident from the nucleotide and protein sequence analysis (Figure 4 and Figure 5). More importantly, non-synonymous nucleotide substitutions were common showing significant separation in accordance with their hosts. Synonymous substitutions of the CP gene reflect parallel evolution. Variations among synonymous nucleotide substitutions follow host codon usage frequency. This indicated that viral genomes evolve alongside host molecular factors. Mutational selection, therefore, may drive microevolution within translational selection forces, especially host codon usage frequency.

## Data Availability

The data are not available due to the nature of this research. Participants of this study did not agree for their data to be shared publicly.

## Supplementary Materials

The following are available online at https://www.mdpi.com/article/10.3390/microorganisms9061111/s1. Figure S1. Percentage nucleotide identities of Korean Apple stem grooving virus (ASGV) coat protein gene using MUSCLE alignment implemented in SDTv1.2. Figure S2. Percentage amino acid identities of Korean apple stem grooving virus (ASGV) coat protein gene using MUSCLE alignment implemented in SDTv1.2. Figure S3. Synonymous (dS) and Non-synonymous (dN) substitution of the apple stem grooving virus (ASGV) coat protein gene (a). Codon usage of ASGV from apple and pear reported sequences from China (b). Figure S4. Secondary structure prediction of Korean apple stem grooving virus (ASGV) coat protein. Amino acid se-quences were rearranged corresponding to the phylogenetic analysis categorization of nucleotide sequences. Boxes rep-resent the significant differences between the two clades from the phylogenetic analysis. Predicted secondary structure results showed similar categorization of nucleotide phylogenetic analysis. Secondary structures were predicted using the original Garnier Osguthorpe Robson algorithm (GOR I) provided by the EMBOSS suite embedded in Geneious Pro 8. Figure S5. Protein model prediction of selected Korean apple stem grooving virus (ASGV) coat protein sequences using i-TASSER. Phylogenetic relationship of the isolates is also shown. The accessions used for model prediction were under-lined. Table S1. List of coat protein gene sequences used in the nucleotide sequence analyses.
