# Supplementary figures and images for: Adaptation and Codon-Usage Preference of Apple and Pear-Infecting Apple Stem Grooving Viruses
Source: Microorganisms. 2021 May 21;9(6):1111. doi: 10.3390/microorganisms9061111 (PMC8223792; doi:10.3390/microorganisms9061111)

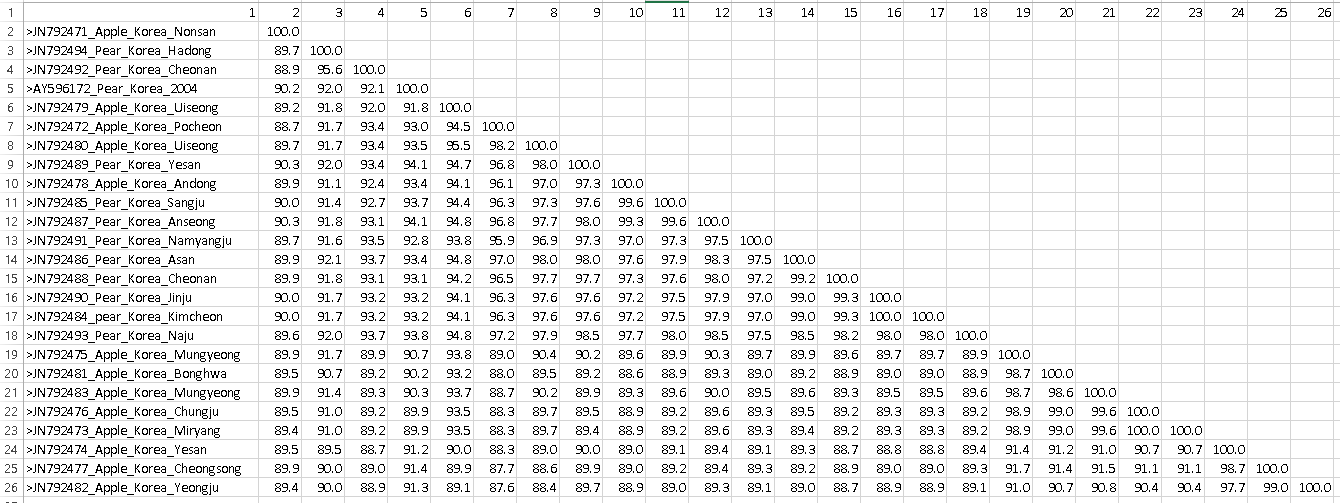

Supplement: Supplementary file 1 [file microorganisms-09-01111-s001.zip › Figure S1.png]

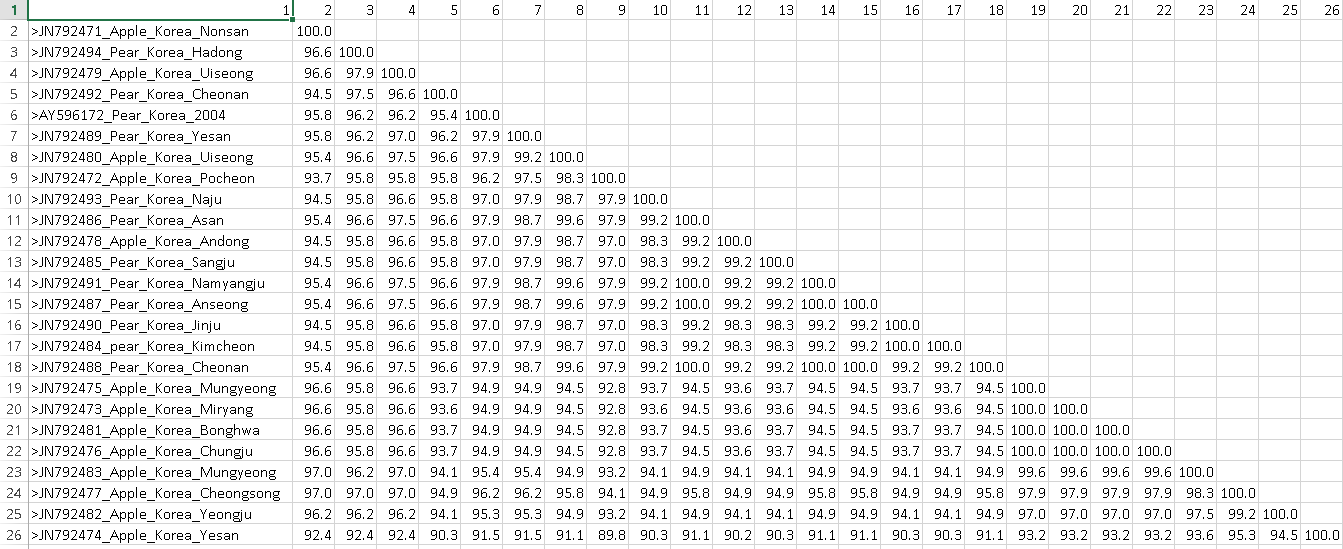

Supplement: Supplementary file 1 [file microorganisms-09-01111-s001.zip › Figure S2.png]

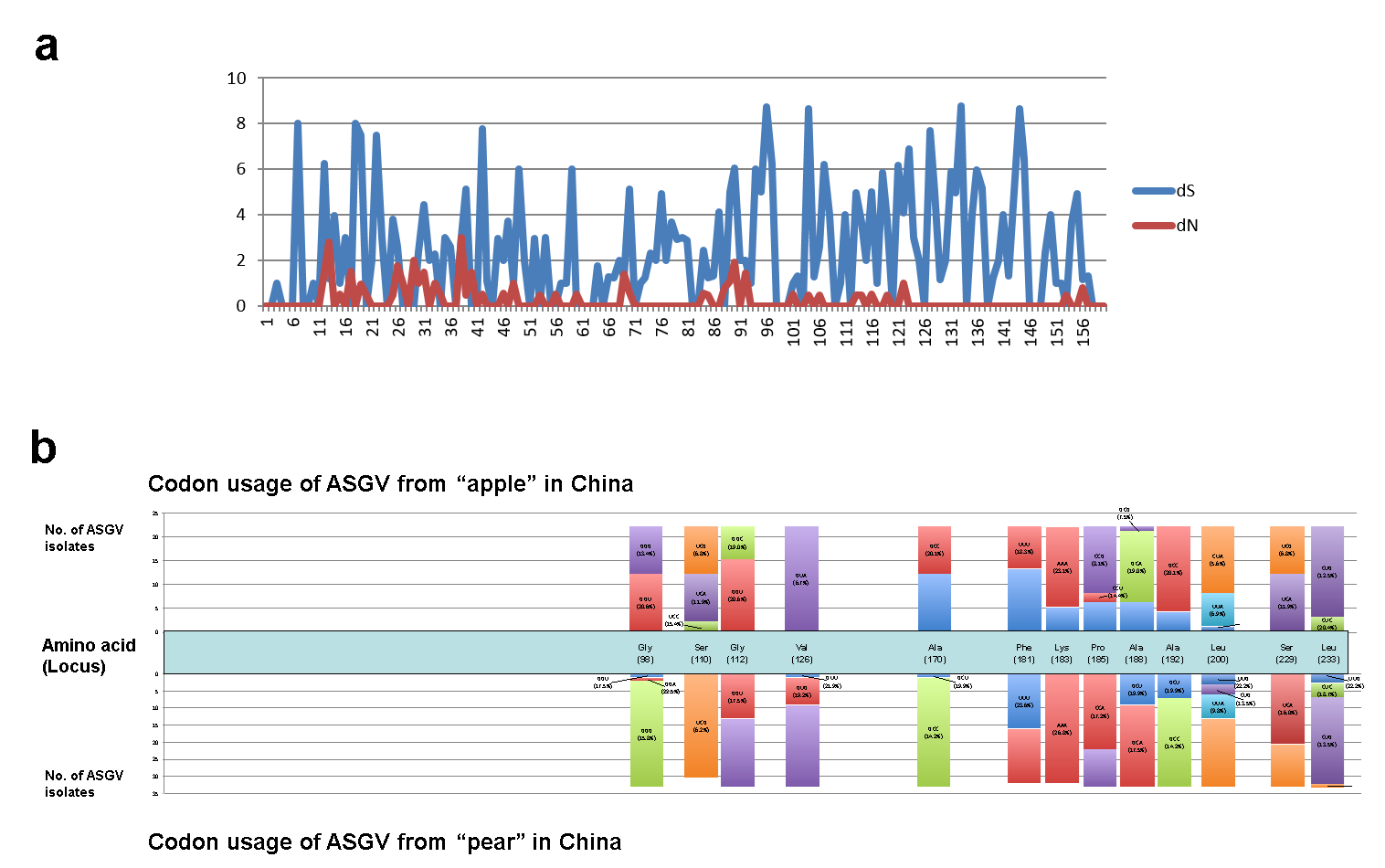

Supplement: Supplementary file 1 [file microorganisms-09-01111-s001.zip › Figure S3.png]

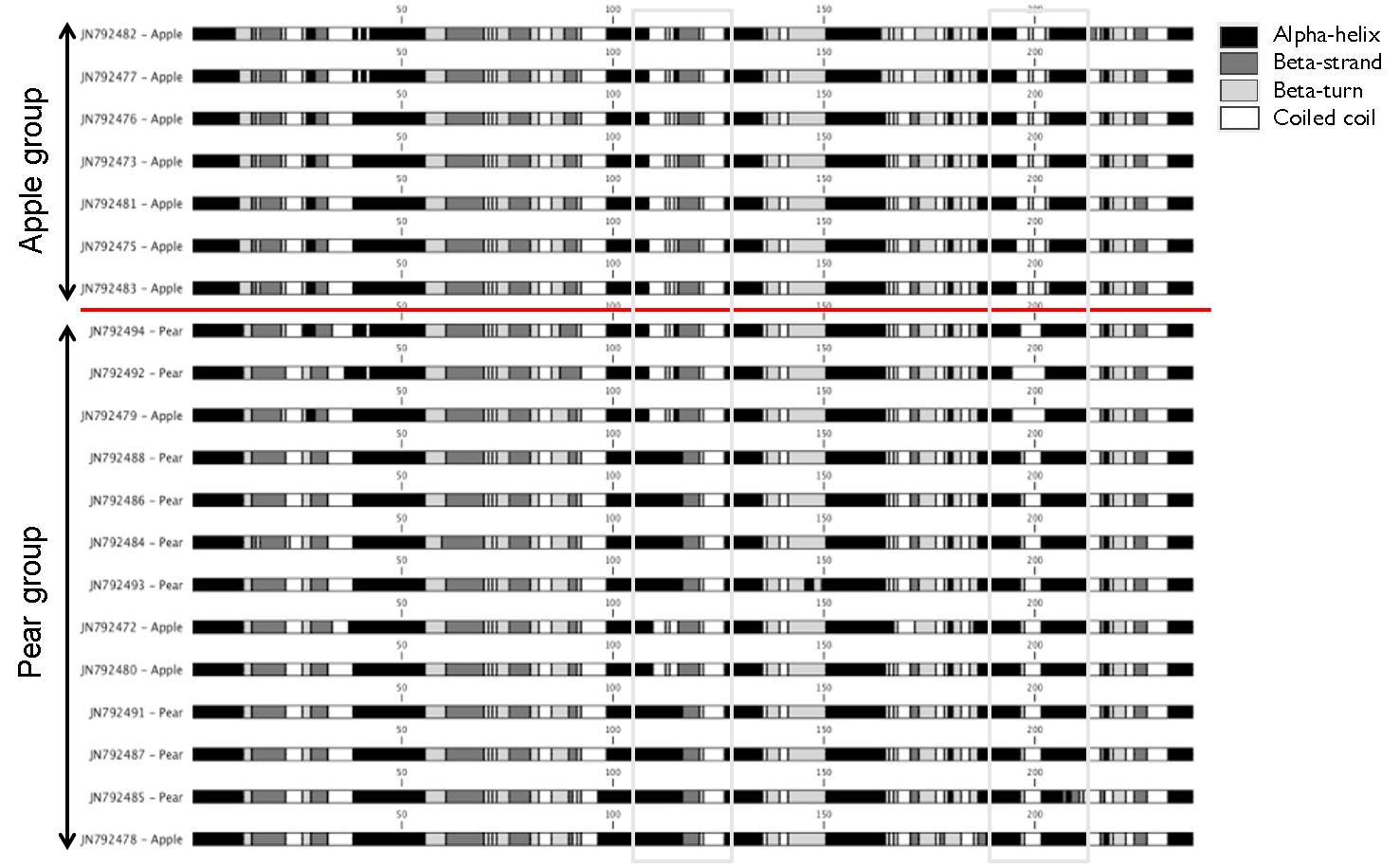

Supplement: Supplementary file 1 [file microorganisms-09-01111-s001.zip › Figure S4.png]

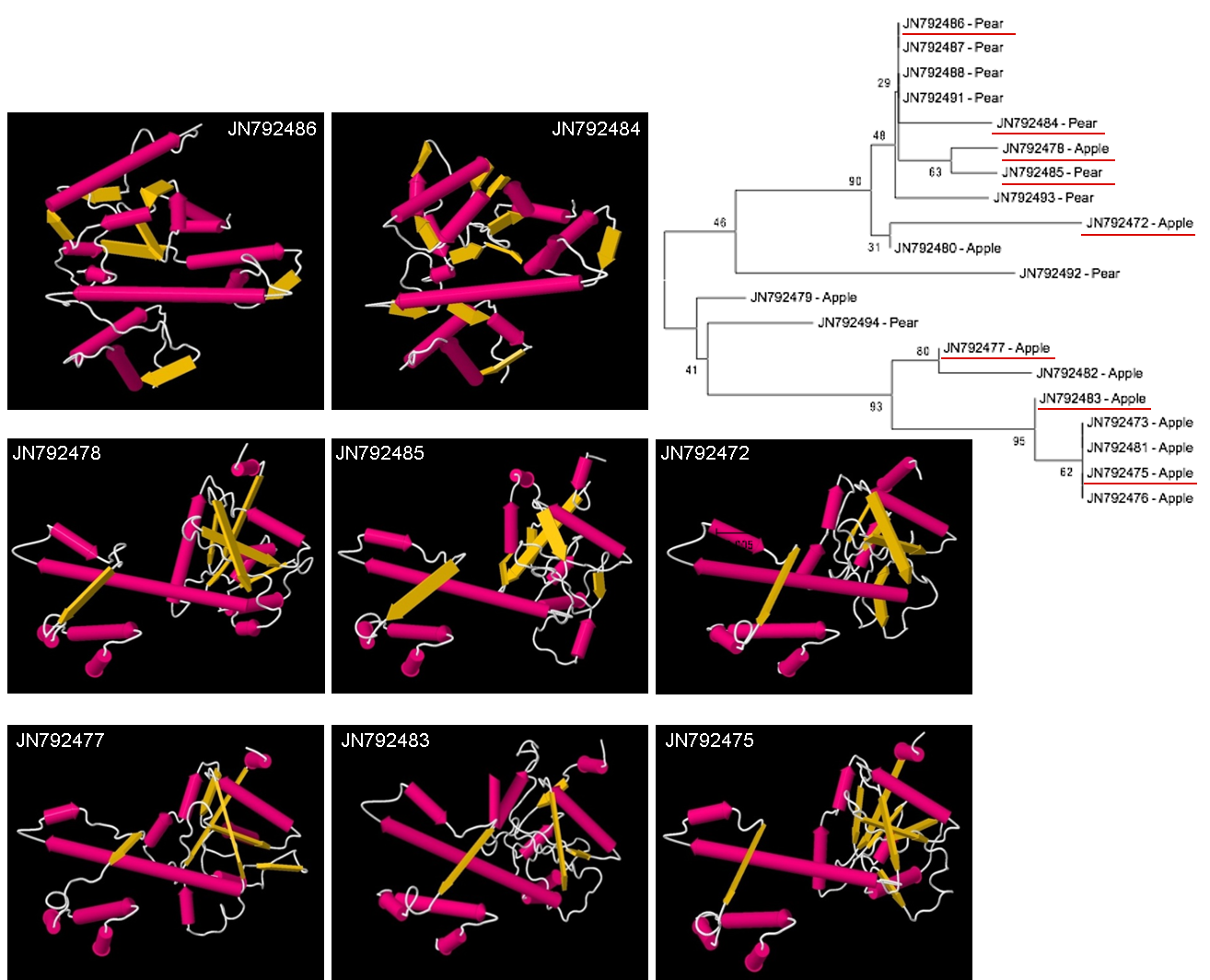

Supplement: Supplementary file 1 [file microorganisms-09-01111-s001.zip › Figure S5.png]
